# Effect of Increased Endometrial Thickness and Implantation Rate by Granulocyte Colony-Stimulating Factor on Unresponsive Thin Endometrium in Fresh In Vitro Fertilization Cycles: A Randomized Clinical Trial

**DOI:** 10.1155/2017/3596079

**Published:** 2017-07-16

**Authors:** Fatemeh Sarvi, Marjan Arabahmadi, Ashraf Alleyassin, Marzieh Aghahosseini, Marzieh Ghasemi

**Affiliations:** ^1^Department of Endocrinology and Infertility, Shariati Hospital, Tehran University of Medical Sciences, Tehran, Iran; ^2^Department of Obstetrics and Gynecology, Pregnancy Health Research Center, Zahedan University of Medical Sciences, Zahedan, Iran

## Abstract

**Background:**

The correlation between endometrial thickness and receptivity has been mentioned in various studies. This study investigated the effect of granulocyte colony-stimulating factor in treating thin endometrium of infertile women who were chosen for in vitro fertilization in our infertility clinic in 2014 and 2015.

**Methods:**

In this randomized clinical trial, 28 women who were chosen for in vitro fertilization and had endometrial thickness of less than 6 mm on the day of human chorionic gonadotropin (hCG) injection were included in the study. They were randomly divided into two groups: investigation and control groups. In investigation group (*n* = 13) one granulocyte colony-stimulating factor vial (300 micrograms in 1 mL) was infused into the uterus within five minutes by embryo transfer catheter. In control group (*n* = 15) 1 mL of saline was injected into the uterus with the same catheter.

**Results:**

There were significant differences between the two groups in terms of means of endometrial thickness on oocyte retrieval day (*P* = 0.001), embryo transfer day (*P* = 0.001), hCG injections (*P* = 0.001), and implantation rates (*P* = 0.001).

**Conclusion:**

Granulocyte colony-stimulating factor can increase endometrial thickness in women treated with in vitro fertilization.* RCT Code* is 201406046063N2.

## 1. Introduction

Endometrial and receptivity factors are important in the success of in vitro fertilization and embryo transfer (IVF-ET) cycles. Many researches have been done to improve it [1–3]. It has been shown that endometrial thickness of less than 7 mm has a negative effect on pregnancy rate [4]. Standard in vitro fertilization (IVF) treatments affect less than 1% of women with thin endometrium. It is a frustrating problem for both patient and physician. It can lead to unwanted cancellation and delay in treatment [5, 6]. The correlation between endometrial thickness and receptivity has been mentioned in various studies [7–10]. However, some studies have not reported such a correlation [11–13]. When endometrium is not appropriately thickened for embryo transfer, the physician uses drugs such as aspirin, sildenafil, pentoxifylline and tocopherol-f. Still, the endometrium remains unresponsive in some cases [14].

In recent years some studies have stated that intrauterine infusion of granulocyte colony-stimulating factor (GCSF) may be effective in patients with treatment-resistant thin endometrium. GCSF is a glycoprotein that affects cytokines and growth factors. Immunological mechanisms in the endometrium are involved in the implantation process. GCSF boosts the endogenous cytokines' secretion and enables various different endocrine routes [15]. In a study Tanaka and colleagues concluded that, in both autocrine and paracrine directions, GCSF results in decidualization of endometrial stromal cells [16].

GCSF stimulates the neutrophilic granulocyte proliferation and is effective on the embryo implantation through decidual cells macrophage activation. Increased Th-2 cytokine secretion and regulatory T cells stimulation are other effects of GCSF. Trophoblastic cells and human luteinized granulose cells express GCSF receptor [17]. Colony-stimulating factors can regulate endometrium's growth. Macrophage colony-stimulating factor is involved in early endometrium development and its effect has been shown on endometrial epithelial cells proliferation [18]. GCSF effects have also been studied in the treatment of recurrent abortion and implantation failure.

Gleicher and colleagues [17] studied the impact of GCSF in increasing endometrial thickness of women whom their previous IVF cycles were cancelled because of thin endometrium. They found that all patients with thin endometrium became pregnant using intrauterine GCSF infusion. Also, in another study on 21 women in 2013, Gleicher and colleagues [19] found that endometrial thickness and pregnancy rate significantly increase after GCSF infusion.

We investigated the effect of intrauterine instillation of GCSF on nonresponsive thin endometrium in women undergoing fresh IVF cycles.

## 2. Materials and Methods

**Figure 1:**
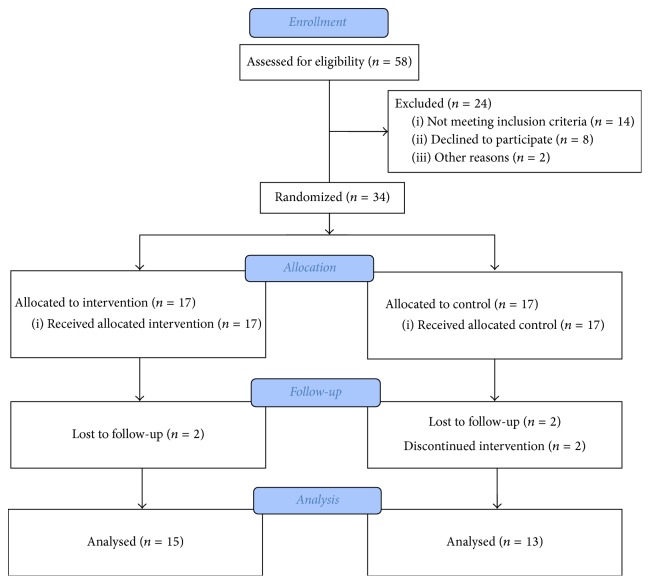
Flowchart of the study.

This parallel randomized clinical trial was done on 34 women who had the inclusion criteria. They were randomly divided into two groups: investigation and control groups (17 women in each group). However, some of the participants did not complete the study and were excluded during the study. Thus, 13 women in investigation group and 15 women in control group completed the study. This study was approved by the ethics committee of Tehran University of Medical Sciences and has been registered in the Iranian Registry of Clinical Trials. The objectives of the study and all treatment interventions were explained to the participants and an informed consent was taken from them before entering the study (Figure 1).

### 2.1. Participants

All treatments were done in our infertility center between May 2014 and May 2015. The inclusion criteria were (1) being an infertile woman who had been chosen for IVF in our center, (2) being 18 to 40 years old, and (3) having at least one previous IVF cycle with a history of thin endometrium unresponsive to treatment. All patients had normal uterine cavity confirmed by hysteroscopy. The exclusion criteria were (1) having any history of surgery on endometrium including curettage or myomectomy, (2) having a history of an autoimmune disease or thrombophilia, (3) having a severe male factor, and (4) having endometrial thickness more than 6 mm on the human chorionic gonadotropin (hCG) trigger day.

### 2.2. Intervention

Transvaginal ultrasound was done on all participants in the early follicular phase. The hormone profile was requested for all of them. The participants underwent long protocol cycles. Oral contraceptive started on the third day of menstrual cycle, followed by gonadotropin-releasing hormone (GnRH) agonist in the mid-luteal phase. Ovarian stimulation with 150–300 IU dosage of gonadotropin (Gonal-f, rFSH, Merk Serono) began from the third day of the next cycle's menstruation. Dosage adjustment was done by assessment of estradiol level and transvaginal ultrasound. Stimulation continued until at least three follicles of 18 mm were seen on ultrasound. Then 10000 IU hCG was injected.

The participants with endometrial thickness less than 6 mm on the day of hCG injection, who were unresponsive to sildenafil and estradiol in previous cycles, were randomly divided into two groups: investigation (15 women) and control groups (13 women). In the investigation group, a dosage of GCSF (300 micrograms in 1 cc) was infused into the uterus by embryo transfer catheter within five minutes. In the control group, 1 cc of normal saline was injected into the uterus with the same type of catheter, and then 10000 units of hCG was intramuscularly injected at the same day for all participants. Oocyte was retrieved 34–36 hours later under transvaginal ultrasound guide and intravenous sedation with low-dosage narcotics such as fentanyl. On the day of puncture, endometrial thickness was measured with transvaginal ultrasound in all participants of both groups. If the endometrial thickness was less than 6 mm in the intervention group, a second dosage of GCSF was injected 2-3 days after oocyte retrieval day. Then 2-3 embryos were transferred on the same day. *β*-hCG was checked after two weeks of embryo transfer. Fetus' heart rate was assessed by transvaginal ultrasound after four weeks.

### 2.3. Outcomes

In both groups, endometrial thickness was measured on the days of hCG trigger, oocyte retrieval, and embryo transfer. GCSF injection was done by one of the researchers at all stages of the project, but ultrasound was done by another researcher who was an infertility subspecialist that had worked for more than eight years in the IVF ward. She was unaware of the study groups and was a blinded observer. Endometrial thickness was measured at the sagittal plane of uterus in transvaginal ultrasound in the most thickened portion.

### 2.4. Randomization and Sequence Generation

Patients were randomly allocated into two groups using a balanced block randomization technique. They were divided into blocks of four. Participants' allocation was done with an online application entitled “Sealed Envelope.”

### 2.5. Sample Size

The sample size was calculated based on endometrial thickness on the embryo transfer day. It was calculated to detect a difference of 2 mm of endometrial thickness between the two groups with 2 mm standard deviation, *α* = 0.05, and power = 80%. Thus, we needed 17 cases in each group.

### 2.6. Statistical Procedure

Data were presented as mean ± standard deviation for continuous variables. Data analysis was done using Stata 13 software through Shapiro-Wilk test, Mann-Whitney *U* test, and independent *t*-tests. *P* value less than 0.05 was considered significant.

## 3. Results

A total of 28 participants completed the study and were included in the analysis. The characteristics including age, body mass index, anti-Mullerian hormone, and mean number of previous IVFs were not significantly different between the two groups (Table 1). Also, previous assisted reproductive technology outcomes were equal between them. There was no significant difference between gonadotropin consumption and known causes of infertility between the two groups (Table 2).

Endometrial thickness on the day of hCG injection was 4.1 ± 1.8 mm in investigation group and 4.2 ± 1.6 mm in control group which was not significant (*P* = 0.8). Endometrial thickness on the day of oocyte retrieval was 8.0 ± 1.0 mm in investigation group and 6.3 ± 1.0 mm in control group which was significant (*P* < 0.001). In the investigation group three women needed a second injection of GCSF, but no women in the control group needed a repeated injection.

Difference in endometrial thickness was significant between the two groups on hCG injection and oocyte retrieval days (*P* < 0.001) (2.1 ± 1.1 mm in control group and 3.9 ± 1.1 mm in investigation group). Endometrial thickness on the day of embryo transfer was 6.9 ± 1.1 mm in control group and 9.1 ± 1.5 mm in investigation group which was significantly different (*P* < 0.001). There was no significant difference between the two groups in terms of number of metaphase II oocytes (*P* = 0.9) (Table 3).

Implantation rate was 5.4% in control group and 10.3% in investigation group which was significant (*P* < 0.001) (Table 4). However, clinical pregnancy rates did not differ (*P* = 0.7) between the two groups (15.3% in investigation group and 20% in control group). There was one case of twin pregnancy in investigation group and none in control group. From among 34 participants at the beginning of study, one woman of control group and two women of investigation group had endometrial thickness more than 6 mm. So they were excluded from the study. One woman in each group did not respond to gonadotropin. Also, a woman was excluded from the investigation group because of refusing to cooperate in follow-up.

## 4. Discussion

Our study showed that GCSF injection increases endometrial thickness. The endometrial thickness averages on oocyte retrieval and embryo transfer days were significantly higher in investigation group. Also, mean of increased endometrial thickness was significantly higher in investigation group.

Wu and colleagues [20] assessed the association between endometrial thickness on the hCG day and IVF-ET outcome in normal responders after GnRH antagonist administration. They found that there is a correlation between endometrial thickness measured on hCG day and clinical outcome. Pregnancy rate was lower in patients with endometrial thickness less than 7 mm compared to patients with endometrial thickness more than 7 mm. In a retrospective study on the clinical data of 756 patients in their first fresh IVF/ICSI cycle, Fang and colleagues [21] investigated the effect of endometrial thickness in hCG day on in vitro fertilization/intracytoplasmic sperm injection (IVF/ICSI) outcome. They concluded that endometrial thickness on the hCG day was associated with pregnancy outcome.

In another study by Gleicher and colleagues [19], means of endometrial thickness were 5.2 mm before GCSF injection, 6.4 mm after injection of first dosage, and 9.3 mm after injection of second dosage; the difference in endometrial thickness after injection was 2.9 mm. Their results are consistent with ours.

In a study on fresh cycles with infused GCSF by Kunicki and colleagues [22], endometrial thickness increased significantly before and after injection in pregnant and nonpregnant women which is consistent with our results. Also, Barad and colleagues [23] investigated whether GCSF affects endometrial thickness, implantation rates, and clinical pregnancy rates in routine, unselected IVF cycles. They concluded that, in normal IVF patients, GCSF does not affect endometrial thickness, implantation rates, or clinical pregnancy rates. Since these results were obtained from an older population than ours, they may not necessarily apply to younger women.

Several studies have been done in this regard in patients with freeze cycles. Bu and colleagues [24] published a retrospective study on the relationship between endometrial thickness on embryo transfer day and pregnancy outcomes in frozen-thawed embryo transfer cycles. They concluded that endometrial thickness on the embryo transfer day significantly affects IVF outcomes in cleavage embryo transfer cycles independent of other factors.

Li and colleagues [25] used low GCSF dosage (100 *μ*g) which had no significant effect on endometrial thickness but had a significant effect on cycle cancellation. This is inconsistent with our findings which might be because of low GCSF dosage in this study and its small sample size. In a prospective study in 2016, Mishra and colleagues [15] investigated 35 women with frozen embryo transfer cycle. They reported that endometrial thickness increased from 5.86 ± 0.58 to 6.58 ± 0.84 after GCSF infusion and 54.28% of them had an increased endometrial thickness more than 7 mm. They concluded that GCSF may increase endometrial thickness but does not improve pregnancy.

A recent study by Xu and colleagues [18] compared the results of GCSF injection and its injection with a scratch of frozen embryo transfer cycle. 30 women randomly received either GCSF or GCSF with scratch. The patients were compared with their previous frozen embryo transfer cycle which was without receiving GCSF. Significantly higher implantation and clinical pregnancy rates were observed in their GCSF group compared to control group (31.5% versus 13.9% and 48.1% versus 25%, resp.). However, live birth rate was not significantly different.

According to Xu and colleagues [18] endometrial thickness significantly increases after GCSF in the same cycle. This is in agreement with our study and Gleicher and colleagues [19] and Kunicki and colleagues [22] studies. Although, in Xu and colleagues' study, there was a self-control group and comparison was done between GCSF and self-control groups, increased endometrial thickness was observed in their intervention group.

In our study, clinical pregnancy rate was 15.3% in the investigation group and 20% in the control group which was not significant. However, the implantation rate was 10.3% in the investigation group and 5.4% in the control group which was statistically significant. In the study of Xu and colleagues [18], significantly higher rates of implantation and clinical pregnancy were observed in their intervention group compared to their control group; they believed that increased clinical pregnancy rate is due to increased endometrial thickness.

Our study was done on fresh cycles, similar to the studies of Gleicher and colleagues [19] and Kunicki and colleagues [22]. Our participants only received GCSF and we had a control group, but Kunicki and colleagues [22] had no control group and their patients received aspirin and sildenafil besides GCSF which can affect the endometrial thickness. However, clinical pregnancy rate in our study was less than Kunicki and colleagues [22] and Gliecher and colleagues [19] studies. Still we could not show the effect of GCSF on clinical pregnancy rate. Mishra and colleagues [15] found increased endometrial thickness after GCSF injection, but, similar to our study, they did not report improvement in clinical pregnancy rate. Also, Eftekhar and colleagues [26] in 2014 failed to show that GCSF improves endometrial thickness. However, they reported that GCSF improves clinical and chemical pregnancy rates.

### 4.1. Limitations

Similar to most other studies on this subject, our study had a small sample size. Also, other mentioned studies were done on fresh and frozen cycles and sometimes they had used drugs such as sildenafil, aspirin, and estrogen. So it is not possible to compare their results with each other.

## 5. Conclusion

This study showed that GCSF might be associated with increased endometrial thickness in women treated with IVF. Also, it can lead to higher implantation rate. However, we did not achieve significantly higher clinical pregnancy rates because of our small sample size. Thus, conducting a study with a larger population and eliminating the confounding factors are recommended.

## Figures and Tables

**Table 1:** Characteristics of participants in both groups.

Variables	Control group (*n* = 15)	Investigation group (*n* = 13)	*P* value
Age (years old)	31.2 ± 3.2	31.6 ± 3.8	0.7
Body mass index (kg/m^2^)	26.6 ± 3.1	26.2 ± 3.5	0.7
Anti-Mullerian hormone	2 ± 1.5	1.9 ± 1.3	0.7
Mean number of previous IVF cycles	1.4 ± 1.2	1.2 ± 1.1	0.7
Mean dosage of used gonadotropins	3360 ± 1201	3629 ± 1078	0.4

**Table 2:** Known causes of infertility in both groups.

Causes	Control group (*n* = 15)	Investigation group (*n* = 13)	*P* value
Diminished ovarian reserve	8 (53%)	5 (38%)	0.41
Ovulatory dysfunction	3 (20%)	4 (30%)	0.53
Male infertility	7 (46%)	5 (38%)	0.66

*Note*. Some women had two reasons for entering IVF cycle treatment.

**Table 3:** Comparing mean outcomes of the studied groups.

Variables	Control group (*n* = 15)	Investigation group (*n* = 13)	*P* value
Day of hCG injection (ET1)	4.2 ± 1.6	4.1 ± 1.8	0.80
Day of oocyte retrieval (ET2)	6.3 ± 1	8 ± 1	0.001
Difference of ET1 and ET2 (Δ)	2.1 ± 1.1	3.9 ± 1.1	0.001
Embryo transfer (ET3)	6.9 ± 1.1	9.1 ± 1.5	0.001
Difference of ET2 and ET3 (Δ)	0.5 ± 0.6	1.1 ± 1	0.11
Mean number of retrieved oocytes	9.2 ± 4.8	9.2 ± 5.3	0.001
Mean number of metaphase II oocytes	6.6 ± 4.5	6.6 ± 5.1	0.99
Difference of endometrial thickness on embryo transfer and hCG injection days	2.6 ± 1.2	5 ± 1.4	0.001

**Table 4:** Rate of implantation and pregnancy (secondary outcomes).

Variables	Control group	Investigation group	*P* value
Implantation rate	5.4%	10.3%	0.001
Clinical pregnancy rate	20%	15.3%	0.7

## References

[1] Cahill D. J., Wardle P. G. (2002). Management of infertility. *British Medical Journal*.

[2] Aaleyasin A., Aghahosseini M., Rashidi M. (2015). In vitro fertilization outcome following embryo transfer with or without preinstillation of human chorionic gonadotropin into the uterine cavity: A randomized controlled trial. *Gynecologic and Obstetric Investigation*.

[3] Alleyassin A., Abiri A., Agha-Hosseini M., Sarvi F. (2017). The value of routine hysteroscopy before the first intracytoplasmic sperm injection treatment cycle. *Gynecologic and Obstetric Investigation*.

[4] Smith J. F., Walsh T. J., Shindel A. W. (2009). Sexual, marital, and social impact of a man's perceived infertility diagnosis. *The Journal of Sexual Medicine*.

[5] Newton C. R., Sherrard W., Glavac I. (1999). The fertility problem inventory: measuring perceived infertility-related stress. *Fertility and Sterility*.

[6] Barker M. A., Boehnlein L. M., Kovacs P., Lindheim S. R. (2009). Follicular and luteal phase endometrial thickness and echogenic pattern and pregnancy outcome in oocyte donation cycles. *Journal of Assisted Reproduction and Genetics*.

[7] Amir W., Micha B., Ariel H., Liat L.-G., Jehoshua D., Adrian S. (2007). Predicting factors for endometrial thickness during treatment with assisted reproductive technology. *Fertility and Sterility*.

[8] Richter K. S., Bugge K. R., Bromer J. G., Levy M. J. (2007). Relationship between endometrial thickness and embryo implantation, based on 1,294 cycles of in vitro fertilization with transfer of two blastocyst-stage embryos. *Fertility and Sterility*.

[9] Zenke U., Chetkowski R. J. (2004). Transfer and uterine factors are the major recipient-related determinants of success with donor eggs. *Fertility and Sterility*.

[10] Kovacs P., Matyas S. Z., Boda K., Kaali S. G. (2003). The effect of endometrial thickness on IVF/ICSI outcome. *Human Reproduction*.

[11] Laasch C., Puscheck E. (2004). Cumulative embryo score, not endometrial thickness, is best for pregnancy prediction in IVF. *Journal of Assisted Reproduction and Genetics*.

[12] Garcia-Velasco J. A., Isaza V., Caligara C., Pellicer A., Remohí J., Simón C. (2003). Factors that determine discordant outcome from shared oocytes. *Fertility and Sterility*.

[13] Dietterich C., Check J. H., Choe J. K., Nazari A., Lurie D. (2002). Increased endometrial thickness on the day of human chorionic gonadotropin injection does not adversely affect pregnancy or implantation rates following in vitro fertilization-embryo transfer. *Fertility and Sterility*.

[14] Casper R. F. (2011). It's time to pay attention to the endometrium. *Fertility and Sterility*.

[15] Mishra V. V., Choudhary S., Sharma U. (2016). Effects of Granulocyte Colony-Stimulating Factor (GCSF) on persistent thin endometrium in Frozen Embryo Transfer (FET) cycles. *Journal of Obstetrics and Gynecology of India*.

[16] Tanaka T., Miyama M., Masuda M. (2000). Production and physiological function of granulocyte colony-stimulating factor in non-pregnant human endometrial stromal cells. *Gynecological Endocrinology*.

[17] Gleicher N., Vidali A., Barad D. H. (2011). Successful treatment of unresponsive thin endometrium. *Fertility and Sterility*.

[18] Xu B., Zhang Q., Hao J., Xu D., Li Y. (2015). Two protocols to treat thin endometrium with granulocyte colony-stimulating factor during frozen embryo transfer cycles. *Reproductive BioMedicine Online*.

[19] Gleicher N., Kim A., Michaeli T. (2013). A pilot cohort study of granulocyte colony-stimulating factor in the treatment of unresponsive thin endometrium resistant to standard therapies. *Human Reproduction*.

[20] Wu Y., Gao X., Lu X. (2014). Endometrial thickness affects the outcome of in vitro fertilization and embryo transfer in normal responders after GnRH antagonist administration. *Reproductive Biology and Endocrinology*.

[21] Fang R., Cai L., Xiong F., Chen J., Yang W., Zhao X. (2016). The effect of endometrial thickness on the day of hCG administration on pregnancy outcome in the first fresh IVF/ICSI cycle. *Gynecological Endocrinology*.

[22] Kunicki M., Łukaszuk K., Woclawek-Potocka I., Liss J., Kulwikowska P., Szczyptańska J. (2014). Evaluation of granulocyte colony-stimulating factor effects on treatment-resistant thin endometrium in women undergoing in vitro fertilization. *BioMed Research International*.

[23] Barad D. H., Yu Y., Kushnir V. A. (2014). A randomized clinical trial of endometrial perfusion with granulocyte colony-stimulating factor in in vitro fertilization cycles: Impact on endometrial thickness and clinical pregnancy rates. *Fertility and Sterility*.

[24] Bu Z., Wang K., Dai W., Sun Y. (2016). Endometrial thickness significantly affects clinical pregnancy and live birth rates in frozen-thawed embryo transfer cycles. *Gynecological Endocrinology*.

[25] Li Y., Pan P., Chen X., Li L., Li Y., Yang D. (2014). Granulocyte colony-stimulating factor administration for infertile women with thin endometrium in frozen embryo transfer program. *Reproductive Sciences*.

[26] Eftekhar M., Sayadi M., Arabjahvani F. (2014). Transvaginal perfusion of G-CSF for infertile women with thin endometrium in frozen ET program: a non-randomized clinical trial. *Iranian Journal of Reproductive Medicine*.

